# Prevalence and sociodemographic correlates of food insecurity among post-secondary students and non-students of similar age in Canada

**DOI:** 10.1186/s12889-023-15756-y

**Published:** 2023-05-25

**Authors:** Yichun Wang, Andrée-Anne Fafard St-Germain, Valerie Tarasuk

**Affiliations:** 1 Dalla Lana School of Public Health, University of Toronto, Toronto, Canada; 2 Department of Nutritional Sciences, Temerty Faculty of Medicine, University of Toronto, 1 King’s College Circle, Toronto, ON M5S 1A8 Canada

**Keywords:** Food insecurity, Young adults, Post-secondary students, Canada

## Abstract

**Background:**

The results of several recent campus-based studies indicate that over half of post-secondary students in Canada are food insecure, but the vulnerability of this group has not been considered in research on predictors of food insecurity in the Canadian population. Our objectives were to (1) compare the prevalence of food insecurity among post-secondary students and non-students of similar age; (2) examine the relationship between student status and food insecurity among young adults while accounting for sociodemographic characteristics; and (3) identify the sociodemographic characteristics associated with food insecurity among post-secondary students.

**Methods:**

Using data from the 2018 Canadian Income Survey, we identified 11,679 young adults aged 19–30 and classified them into full-time postsecondary students, part-time post-secondary students, and non-students. Food insecurity over the past 12 months was assessed with the 10-item Adult Scale from the Household Food Security Survey Module. Multivariable logistic regression analyses were used to estimate the odds of food insecurity by student status while accounting for sociodemographic characteristics, and to identify sociodemographic characteristics predictive of food insecurity among post-secondary students.

**Results:**

The prevalence of food insecurity was 15.0% among full-time postsecondary students, 16.2% among part-time students, and 19.2% among non-students. After adjusting for sociodemographic factors, full-time postsecondary students had 39% lower odds of being food insecure as compared to non-students (aOR 0.61, 95% CI 0.50–0.76). Among postsecondary students, those with children (aOR 1.93, 95%CI 1.10–3.40), those living in rented accommodation (aOR 1.60, 95%CI 1.08–2.37), and those in families reliant on social assistance (aOR 4.32, 95%CI 1.60-11.69) had higher adjusted odds of food insecurity, but having at least a Bachelor’s degree appeared protective (aOR: 0.63, 95% CI 0.41–0.95). Every $5000 increase in adjusted after-tax family income was also associated with lower adjusted odds of food insecurity (aOR 0.88, 95%CI 0.84–0.92) among post-secondary students.

**Conclusions:**

In this large, population-representative sample, we found that young adults who did not attend post-secondary school were more vulnerable to food insecurity, particularly severe food insecurity, than full-time post-secondary students in Canada. Our results highlight the need for research to identify effective policy interventions to reduce food insecurity among young, working-age adults in general.

**Supplementary Information:**

The online version contains supplementary material available at 10.1186/s12889-023-15756-y.

## Background

Household food insecurity is recognized as a serious public health issue in Canada, affecting an estimated 5.8 million people [1]. Systematically monitored since 2005, the socio-demographic and geographic correlates of food insecurity in Canada are well documented. Food insecurity is most prevalent among households with inadequate and insecure incomes and few assets [2–6], with risk greatest among lone-parent families, social assistance recipients, individuals who identify as Indigenous or Black, and people who live in Nunavut [2, 7]. However, the recent proliferation of food insecurity research on university campuses suggests extreme levels of vulnerability among post-secondary students – a group whose vulnerability has not been considered in analyses of food insecurity monitoring data in Canada. Similar to findings in the US [8–10], several campus-based surveys in Canada have reported rates of food insecurity that are several times higher than population prevalence estimates [11–19]. While differences in survey designs, sampling frames, response rates, and food insecurity measurements preclude direct comparisons between these studies and population monitoring data, the magnitude of the differences in prevalence is perplexing. Most recently, a survey of 6167 students on 13 university campuses conducted in Fall 2021 found that 56.8% of students were moderately or severely food insecure and noted a sharp increase from the prevalence charted in a 2016 survey [16]. The comparable population prevalence of moderate or severe household food insecurity in 2021 was 11.2% [20].

Understanding the scale and severity of food insecurity among post-secondary students in Canada is important given studies linking this condition to dietary compromises [21], poorer mental health [12, 13, 19, 22], poorer overall health [10, 12, 13], and poorer academic achievement [10, 12, 13, 21] among students. Many universities have implemented campus food banks, community gardens, and subsidized dining locations to combat food insecurity, but the high estimates of food insecurity among post-secondary students have also prompted calls for broader scale policy interventions including tuition supports, food subsidies [12, 13, 16] and a basic income program [23]. Yet, the absence of any systematic examination of food insecurity among post-secondary students in Canada through the lens of population-representative surveys limits understanding of how the problem documented through campus-based studies relates to the broader population health problem of food insecurity in this country.

Drawing on population-representative survey data for Canada, this study was undertaken to (1) compare the prevalence of food insecurity among post-secondary students and non-students of similar age; (2) examine the relationship between student status and food insecurity among young adults, while accounting for sociodemographic characteristics; and (3) identify the sociodemographic characteristics associated with food insecurity among post-secondary students.

## Methods

### Data source and study population

This study used data from the 2018 Canadian Income Survey (CIS). CIS is an annual cross-sectional survey administered by Statistics Canada and designed to provide information on the income, income sources, and sociodemographic characteristics of Canadians. The CIS is administered to a subsample of respondents in the Labor Force Survey (LFS) interviews, with the data from the CIS survey interviews supplemented with information from the LFS and income tax [24]. Generally, a knowledgeable member of the household provided the LFS information for all members of the household and the CIS information for members aged 16 and older, including questions related to food insecurity. The survey was conducted between January and June, 2019, by telephone interviews, personal visits, or online questionnaire. The overall response rate to CIS 2018 was 77.4% [24]. Although data were collected nationally, only data for the ten provinces were available at the time of this study.

We limited this study to young adults aged 19 to 30 years who were members of households sampled in CIS 2018. This age range was selected to maximize the probability of including post-secondary students and excluding young adults who are still attending high school. Although most Canadians have completed high school by 19 years of age [25], we further screened for indications of high school attendance, excluding individuals who reported attending school in 2018 but had not completed high school and whose highest level of education was less than high school completion. The final sample was 11,679.

### Measures

Household food insecurity over the previous 12 months was assessed using the Household Food Security Survey Module (HFSSM). As noted above, one household member responded to this module on behalf of the entire household; in multi-adult households, the respondent may or may not have been the young adult included in our sample. We determined young adults’ food insecurity status from the 10-item adult subscale of the HFSSM, consistent with prior Canadian studies of post-secondary students that have used this module [11, 13, 17, 18]. Adult food insecurity status was defined as food secure (no affirmative responses), marginally food insecure (1 affirmative response), moderately food insecure (2–5 affirmative responses), and severely food insecure (≥ 6 affirmative responses) [26]. To prevent small cell counts, all regression models for objectives 2 and 3 were conducted on the binary variable (food secure vs. food insecure), aggregating marginal, moderate and severe food insecurity.

Post-secondary student status identified non-students, part-time students, and full-time students based on whether individuals attended school in 2018 and whether this attendance was part-time or full-time. The determination of post-secondary school attendance on this survey encompassed public and private universities and colleges and Collèges d’enseignement général et professionnel (CEGEPs) but did not differentiate between these institutions. The sociodemographic characteristics were identified based on prior research on the correlates of food insecurity in the general population and among post-secondary students [2–7, 11, 16, 17]. These included the individual’s age group, sex, immigration status, Indigenous status, highest level of education achieved, living arrangements, as well as province and size of the area of residence, major source of income of the economic family, homeownership, and adjusted, annual after-tax income of the economic family. Statistics Canada imputed missing data for these variables using nearest neighbor approach and deterministic imputation [24].

Economic family is defined by Statistics Canada as individuals who live in the same dwelling and are related to each other by blood, marriage, common-law, adoption, or a foster relationship [24]. Living arrangement was classified using economic family type and the individual’s relationship to the major income earner in the economic family. The categories of living arrangement included living alone with or without roommates, with family (parents or relatives), with partner only, or with children. The last category grouped couples with children and lone-parent families due to small cells.

Considering that many young adults lived with parents, relatives, a partner and/or children, the after-tax income of the economic family was used to account for the nature of shared resources and living expenses among family members. The income was adjusted for family size by dividing the family’s after-tax income by the square root of family size [27]. The major income source of the economic family identified whether the economic family was primarily reliant on employment income (wages, salaries, or self-employment), social assistance, or other sources. Homeownership assessed whether the dwelling was owned by a member of the economic family.

The affordability of post-secondary schooling differs by province, and this could affect students’ risk of food insecurity. To adjust for this potential source of variation, we adapted the Canadian Centre for Policy Alternatives’ “Cost of Learning Index” [28] to classify provinces into one of three categories of affordability of a university education (low, medium or high). This relative measure of affordability considers university tuitions and compulsory ancillary fees in relation to family incomes [28]. We updated the index published in 2013 to account for subsequent tuition policy changes implemented by provincial governments with respect to in-province students attending universities, as well as annual inflation of incomes between 2013 and 2018 (Additional file 1).

### Statistical analysis

The prevalence of marginal, moderate, and severe food insecurity was estimated among non-students, part-time, and full-time students with 95% confidence intervals. Proportions and means were used to describe the sociodemographic characteristics of food-secure and food-insecure non-students, part-time students, and full-time students.

Logistic regressions were run on each covariate against the binary outcome of food insecurity to generate unadjusted odds ratios. Multivariable logistic regression models were then run to produce adjusted odds ratios of food insecurity by student status while accounting for demographic, geographic, and economic characteristics. We built a two-stage model, first adjusting only for the demographic and geographic characteristics of the individual (age group, sex, immigration and Indigenous status, education level, living arrangements, and province and size of area of residence), and then adding variables describing the material circumstances of the individual’s economic family (family after-tax income, major income source, and homeownership). Since economic factors are major predictors of food insecurity, this approach allowed us to observe how accounting for them influenced the relationships between food insecurity and the student status and demographic characteristics of the individuals. For all categorical predictors, the category with the most observations was the reference group. Inspection of the Tolerance and Variance Inflation Factor revealed no indication of multicollinearity in the adjusted models.

We identified the predictors of food insecurity among part-time and full-time post-secondary students, first estimating unadjusted odds ratios and then applying a multivariable logistic regression model including the aforementioned demographic, geographic and economic characteristics. We included two additional variables, employment status (full-time/part-time/did not work during the reference year) and receipt of scholarship (yes/no).

All analyses were conducted with SAS version 9.4, using SURVEY commands with the individual-level survey weights to calculate population-based estimates and bootstrap weights provided by Statistics Canada.

## Results

**Fig. 1:**
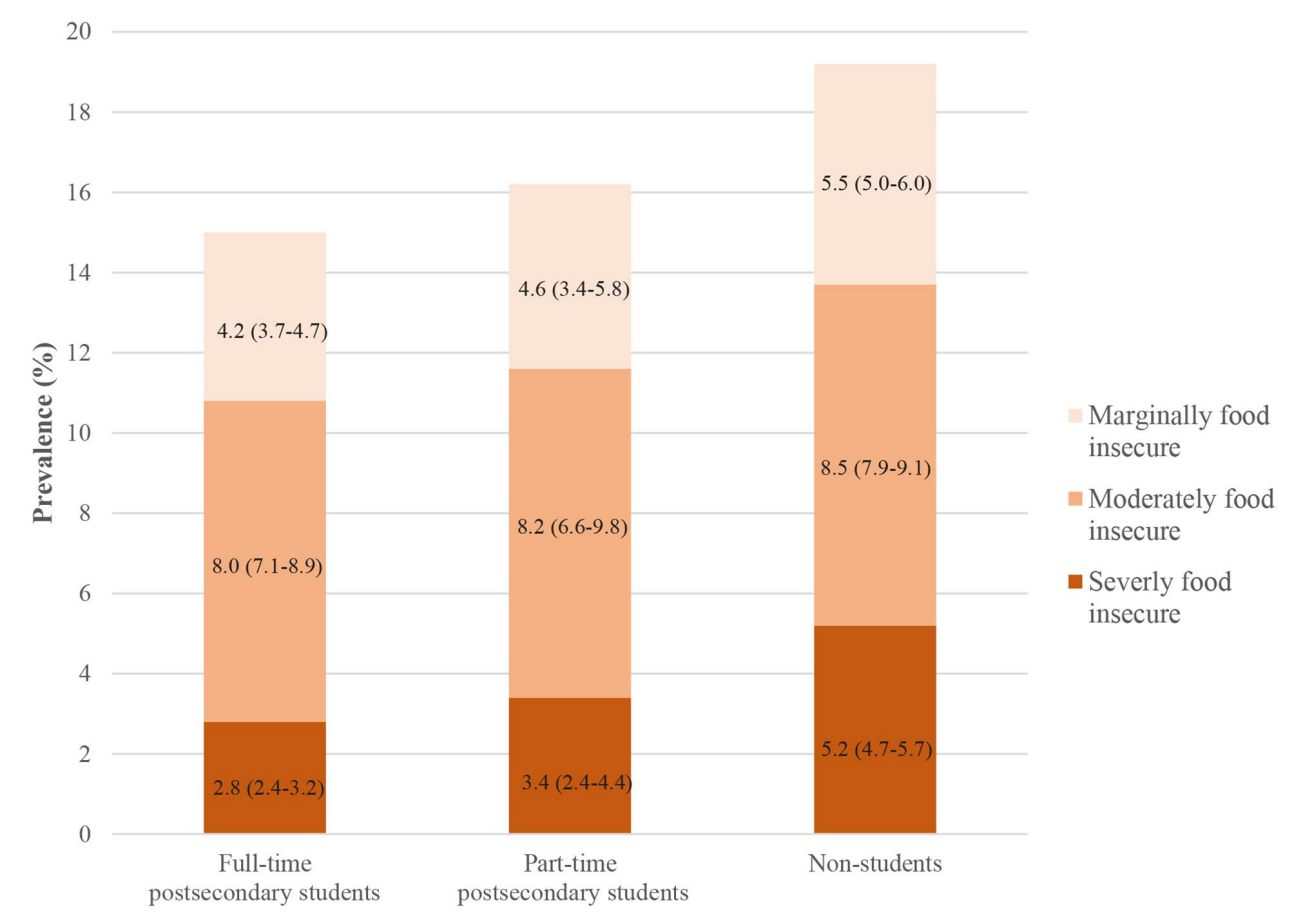
Prevalence (95% Confidence Intervals) of marginal, moderate, and severe food insecurity by student status

Among the study population of young adults, 29.7% were full-time post-secondary students, 5.5% were part-time post-secondary students, and 64.9% were not students. The prevalence of food insecurity overall was 15.0% among full-time students, 16.2% among part-time students, and 19.2% among non-students (Fig. 1). The greatest difference between groups was in the prevalence of severe food insecurity; 5.2% (95% CI 4.7–5.7%) of non-students were severely food insecure compared to 3.4% (95% CI 2.4–4.4%) and 2.8% (95% CI 2.4–3.2%) for part-time and full-time students, respectively. The distribution of food insecurity status by socio-demographic characteristics, stratified by student status, is presented in Additional File 2.

Full-time post-secondary students had 39% lower adjusted odds of food insecurity compared to non-students (aOR 0.61, 95% CI 0.50–0.76) once we adjusted for individual socio-demographic and geographic characteristics and family-level economic factors (Table 1). Neither the crude nor adjusted odds of food insecurity among part-time students compared to non-students were statistically significant.

Other characteristics associated with significantly lower odds of food insecurity among young adults in our fully adjusted model include having a Bachelor’s degree or above, living alone or with a spouse but no children, and having a higher family income (Table 1). The odds of food insecurity were significantly elevated for young adults who were Indigenous, those in families reliant on social assistance, and those living in rented accommodation (Table 1).

**Table 1:** Crude and adjusted odds of food insecurity in students (full- and part-time) as compared to non-students by demographic categories, Canada, 2018 (n = 11,679)

	Crude OR (95% CI)	OR (95% CI), partially adjusted^1^	OR (95% CI), fully adjusted^2^
**Student Status**			
Part time student	0.82 (0.61–1.10)	0.88 (0.65–1.18)	0.90 (0.64–1.25)
Fulltime student	0.75 (0.62–0.90)**	0.72 (0.58–0.89)**	0.61 (0.50–0.76)***
Non-student	1.00	1.00	1.00
**Provincial ranking of affordability of post-secondary schooling**			
High affordability (Newfoundland and Labrador, Quebec, Manitoba)	0.98 (0.77–1.24)	0.94 (0.73–1.21)	0.89 (0.68–1.16)
Medium affordability (Prince Edward Island, New Brunswick, Alberta, British Columbia)	1.15 (0.94–1.42)	1.05 (0.84–1.31)	1.14 (0.90–1.44)
Low affordability (Nova Scotia, Ontario, Saskatchewan)	1.00	1.00	1.00
**Age group**			
19–24	1.13 (0.96–1.32)	1.11 (0.92–1.36)	1.06 (0.87–1.28)
25–30	1.00	1.00	1.00
Sex			
Male	1.00	1.00	1.00
Female	1.09 (0.96–1.23)	1.18 (1.04–1.35)*	1.12 (0.97–1.28)
**Immigration status**			
Canadian born	1.00	1.00	1.00
Immigrant ≤ 5 years	1.16 (0.83–1.62)	1.38 (0.96–1.98)	0.96 (0.65–1.42)
Immigrant > 5 years	0.85 (0.65–1.11)	0.98 (0.73–1.31)	0.84 (0.62–1.13)
**Aboriginal Status**			
Non-aboriginal	1.00	1.00	1.00
Aboriginal	2.48 (1.84–3.33)***	1.94 (1.43–2.63)***	1.52 (1.12–2.05)**
**Highest level of education**			
High school or less	1.61(1.35–1.92)***	1.63 (1.34–1.98)***	1.19 (0.97–1.46)
Some post-secondary education (no certificate)	1.30 (0.99–1.69)	1.52 (1.15–2.01)**	1.23 (0.91–1.65)
Post-secondary certificate below Bachelor’s	1.00	1.00	1.00
Bachelor’s degree or above	0.52 (0.41–0.66)***	0.49 (0.39–0.63)***	0.53 (0.41–0.69)***
**Size of area of residence**			
Rural area/ population < 100,000	1.00	1.00	1.00
Population 100,000- 499,999	0.99 (0.81–1.22)	1.11 (0.89–1.38)	0.96 (0.75–1.22)
Population ≥ 500,000	0.86 (0.71–1.05)	1.09 (0.88–1.36)	0.92 (0.73–1.17)
**Living arrangements**			
Living alone with/without roommates	1.38 (1.08–1.76)*	1.70 (1.29–2.22)**	0.59 (0.43–0.80)**
Living with families (parents or relatives)	1.00	1.00	1.00
Living with spouse, no children	0.83 (0.63–1.08)	1.05 (0.78–1.41)	0.68 (0.50–0.92)*
Living with children	2.03 (1.63–2.52)***	2.10 (1.62–2.73)***	1.08 (0.82–1.43)
**Economic family: total adjusted income after tax (Mean ± SE)**	0.88 (0.86–0.90)***	NA	0.88 (0.85–0.90)***
**Major source of economic family income, %**			
Wages, salaries, or self-employment	1.00	NA	1.00
Social assistance	14.42 (8.89–23.38)***	NA	4.36 (2.60–7.31)***
Other income sources	1.59 (1.24–2.04)**	NA	0.87 (0.64–1.16)
**Ownership of dwelling, %**			
Owned by a member of the household	1.00	NA	1.00
Not owned by a member of the household	2.31 (1.91–2.81)***	NA	1.52 (1.21–1.91)**

^1^The partially adjusted model includes demographic and geographic characteristics of the individual (age group, sex, immigration and Indigenous status, education level, living arrangements, and province and size of area of residence)

^2^The fully adjusted model includes the demographic and geographic characteristics included in the partially adjusted model plus variables describing the material circumstances of the economic family of the individual (family after-tax income, major income source, and homeownership)

* p value < 0.05, ** p value < 0.01, *** p value < 0.0001

Among post-secondary students, those with children (aOR 1.93, 95%CI 1.10–3.40), those living in rented accommodation (aOR 1.60, 95%CI 1.08–2.37), and those in families primarily reliant on social assistance (aOR 4.32, 95%CI 1.60-11.69) had higher adjusted odds of food insecurity (Table 2). Compared to students with some post-secondary education, students who had bachelor’s degrees or above had lower adjusted odds of food insecurity (aOR: 0.63, 95% CI 0.41–0.95). Every $5000 increase in adjusted after-tax family income was also associated with 12% lower adjusted odds of food insecurity (aOR 0.88, 95%CI 0.84–0.92). The unadjusted odds of food insecurity were 2 times as high for Indigenous (OR 2.07, 95%CI 1.29–3.30) and recent immigrant (OR 2.05, 1.23–3.41) students, but adjustment for other sociodemographic and economic characteristics resulted in statistically non-significant odds in both groups. The unadjusted and adjusted odds of food insecurity did not differ significantly by part- versus full-time student status or employment status, age, sex, receipt of scholarship, the relative affordability of a university education in the province, or the population density of the area of residence.

**Table 2:** Crude and adjusted odds of food insecurity among post-secondary students by demographic categories, Canada, 2018 (n = 4,102)

	Crude OR (95% CI)	Adjusted^1^ OR (95% CI)
**Student Status**		
Part time post-secondary student	1.09 (0.79–1.52)	1.32 (0.87–1.99)
Full time post-secondary student	1.00	1.00
**Provincial ranking of affordability of post-secondary schooling**		
High affordability (Newfoundland and Labrador, Quebec, Manitoba)	1.08 (0.72–1.61)	1.05 (0.65–1.70)
Medium affordability (Prince Edward Island, British Columbia, Alberta, New Brunswick)	1.00 (0.73–1.38)	1.00 (0.68–1.46)
Low affordability (Nova Scotia, Ontario, Saskatchewan)	1.00	1.00
**Age groups**		
19–24 years	0.85 (0.64–1.13)	0.87 (0.61–1.25)
25–30 years	1.00	1.00
**Sex**		
Male	1.00	1.00
Female	1.16 (0.92–1.47)	1.08 (0.84–1.40)
**Immigration status**		
Canadian born	1.00	1.00
Immigrant ≤ 5 years	2.05 (1.23–3.41)**	1.20 (0.66–2.21)
Immigrant > 5 years	1.11 (0.75–1.64)	0.94 (0.59–1.48)
**Aboriginal Status**		
Non-aboriginal	1.00	1.00
Aboriginal	2.07 (1.29–3.30)**	1.40 (0.86–2.27)
**Highest level of education**		
High school degree	1.23 (0.87–1.72)	1.30 (0.86–1.97)
Some post-secondary education (no certificate)	1.38 (0.97–1.99)	1.39 (0.90–2.16)
Post-secondary certificate below Bachelor’s	1.00	1.00
Bachelor’s degree or above	0.72 (0.50–1.02)	0.63 (0.41–0.95)*
**Received scholarship**		
Yes	1.23 (0.90–1.68)	1.22 (0.86–1.74)
No	1.00	1.00
**Size of area of residence**		
Rural area/ population < 100,000	1.00	1.00
Population 100,000- 499,999	1.40 (0.99–1.99)	1.14 (0.76–1.72)
Population ≥ 500,000	1.18 (0.86–1.62)	0.97 (0.67–1.38)
**Living arrangements**		
Living alone with/without roommates	1.73 (1.12–2.68)*	0.57 (0.31–1.03)
Living with families (parents or relatives)	1.00	1.00
Living with spouse, no children	1.37 (0.87–2.16)	0.79 (0.45–1.37)
Living with children	4.25 (2.70–6.69)***	1.93 (1.10–3.40)*
**Employment**		
Full-year full-time worker	0.98 (0.68–1.39)	1.04 (0.66–1.65)
Part-time worker	1.00	1.00
Did not work during the reference year	1.39 (1.02–1.89)*	0.94 (0.63–1.41)
**Household income**		
Total adjusted income after tax (Every 5000 CAD)	0.88 (0.86–0.91)***	0.88 (0.84–0.92)***
**Major source of economic family income**		
Wages, salaries, or self-employment	1.00	1.00
Social assistance	13.00 (5.31–31.83)***	4.32 (1.60-11.69)**
Other income sources	1.73 (1.19–2.51)**	0.92 (0.58–1.46)
**Ownership of dwelling**		
Owned by a member of the household	1.00	1.00
Not owned by a member of the household	2.88 (2.14–3.88)***	1.60 (1.08–2.37)*

^1^The adjusted model includes all of the variables listed in this table

* p value < 0.05, ** p value < 0.01, *** p value < 0.0001

## Discussion

In this large, population-based sample of young adults, full- and part-time post-secondary students had slightly lower prevalence of food insecurity, especially severe food insecurity, than non-students. Contrary to the conclusions drawn from several recent campus-based surveys [11–19], we found no evidence that post-secondary students were disproportionately affected by food insecurity. Compared to young adults who were not students, being a full-time post-secondary student was associated with significantly lower odds of food insecurity, even after taking into account individual characteristics and material circumstances.

Among post-secondary students, food insecurity was closely linked to students’ material circumstances, as indicated in this study by family income, housing tenure, and family reliance on social assistance. This finding is consistent with the results of more in-depth studies of post-secondary students’ experiences of food insecurity [23]. The higher odds of food insecurity among students who lived with children has also been repeatedly observed in campus-based surveys [11, 16], and it highlights the heightened vulnerability of students who are parents. Our finding that there was no significant difference in the odds of food insecurity by full- versus part-time status is also consistent with prior campus-based studies [11–13]. We lacked the information to classify students according to their degree program or year of study, but our finding of lower odds of food insecurity among students who already had at least a Bachelor’s degree is consistent with other research suggesting that graduate students and those in medical school are at lower risk [11], perhaps because these students have better access to financial support.

Although several campus-based studies have documented an elevated risk of food insecurity among Indigenous and international students [11, 12, 14, 16, 17, 29], we found no significant association between Indigeneity or recent immigration and food insecurity once adjusting for several other characteristics. This suggests that the heightened vulnerability to food insecurity among students who were Indigenous or recent immigrants in our study sample was strongly influenced by other demographic and economic factors.

Whereas most Canadian studies of post-secondary students have been conducted with university students, our identification of post-secondary students included those attending universities, colleges, and CEGEPs, and we were unable to differentiate these groups. This broader definition of post-secondary students may explain why a higher proportion of students in our sample were living with family and engaged in full- or part-time work, compared to some campus-based surveys [11, 16, 1,7]. How the inclusion of CEGEP, college and university students affected our food insecurity prevalence estimates is unclear. In the US, higher rates of food insecurity have been documented among post-secondary students attending two- versus four-year programs [30], suggesting that students in colleges and CEGEP may be more vulnerable than university students, but similar comparisons have not been conducted in Canada yet.

The prevalence of food insecurity charted among post-secondary students in this study is much lower than rates reported from recent campus-based surveys [11–19]. One part of the explanation for this discrepancy may be the different measures of food insecurity used in some studies [12, 14, 16], but even campus-based surveys using the adult scale of the HFSSM or validated shorter forms of this module have yielded much higher prevalence estimates that we observed [11, 13, 15, 17, 19]. A growing body of evidence suggests that some post-secondary students interpret and respond to questions about food insecurity differently than the general population [9], so even when identical questionnaires are used, the results may not be comparable. Confusion may arise because the references to ‘household’ and financial constraints on standardized questionnaires may not reflect the living circumstances and resource constraints of relevance to students living on campuses [9]. It is also important to note that the campus-based surveys cited here had response rates ranging between 2% and 44% [11–15, 17–19]. With such low response rates, the samples are unlikely to be representative of the entire student population on these campuses; participation may be biased towards students who are more concerned about food access issues.

The significantly higher prevalence of severe food insecurity among non-students compared to students is important given the well-documented associations between severe food insecurity and morbidity and premature mortality in Canada [31–33]. Our sample was not large enough to support further interrogation of this finding, but more research is warranted to determine the individual and family circumstances that predispose young adults to severe food insecurity, given the profound negative health implications of this condition.

In interpreting our results, it is important to note that the young adults who comprise our sample were not necessarily the respondents to the HFSSM. This household-level measure was completed by one person in the household on behalf of all members. Young adults living with family members or relatives may have been less likely to be identified as the most knowledgeable household member to respond to the HFSSM. With 69% of post-secondary students in our sample living with family members or relatives, their food insecurity status could be misrepresented when someone else in the household responded to the HFSSM, although the direction of the bias is unknown. An additional limitation of this ‘proxy reporting’ is that the student may have been living away from home for part of the year and the person responding to the HFSSM may have been unaware of the student’s experience of food insecurity when absent from the family home. Related to this, we had no way to identify students living on campuses or students who were financially independent from their parents – a variable highly correlated with student food insecurity in other research [10]. We cannot gauge how much our use of a household-level measure of food insecurity potentially completed by a family member other than the young adult in our sample may have biased our results. Conducting a similar analysis with data from the 2014–2018 Current Population Survey in the US, Gundersen [8] determined that the food insecurity prevalence among college students whose food insecurity was reported by a parent or caregiver was not materially different from the prevalence among students who completed the HFSSM themselves. Whether this is similarly true in Canada remains to be determined.

Our study is further limited by our lack of data to examine food insecurity rates among the broad array of indicators of marginalization (e.g., sexual identity, disability) that have been identified as predictors of food insecurity among university students [14, 16].

We attempted to account for provincial differences in the affordability of university education by grouping provinces based on the “Cost of Learning” index [28], but this categorical variable was not a significant predictor of food insecurity in any of our models. One limitation of the index is that it did not account for costs associated with attending colleges or CEGEPs, which were also included in our study. More research is needed to identify the provincial and federal policies of most relevance to young adults in post-secondary education.

Finally, it should be noted that our analysis was conducted on population survey data collected before the onset of the COVID-19 pandemic in Canada. The population prevalence of household food insecurity has remained relatively stable through subsequent years of CIS [1], but whether the relationship between post-secondary student status and risk of food insecurity has changed over this period is unknown.

In conclusion, by comparing post-secondary students to non-students of similar age, our study suggests that attending post-secondary school is not a risk factor for food insecurity among young adults in Canada. To the contrary, young adults who do not attend post-secondary school appear more vulnerable to food insecurity, particularly severe food insecurity, than full-time post-secondary students. While previous campus-based studies have called for strategies and policies to address food insecurity among post-secondary students specifically [12, 13, 16], our population-based analysis highlights the need to develop effective interventions to address food insecurity among young adults in general. Studies have shown that income-based interventions, including child benefits [34–36] and public pension programs for seniors [37], reduced household food insecurity in Canada, but more research is needed to understand the most effective policy mechanisms to reduce food insecurity among young, working-age adults.

## Electronic supplementary material

Below is the link to the electronic supplementary material.

Supplementary Material 1

Supplementary Material 2

## Data Availability

The data that support the findings of this study are accessible through Statistics Canada, but restrictions apply to data access. For the current study, the data were accessed under contract through the Statistics Canada Research Data Centre at the University of Toronto, and so are not publicly available. The data are available only with the permission of Statistics Canada. Inquiries regarding data access should be sent to the Canadian Research Data Centre Network at info@crdcn.ca.
