# Supplementary material for: Prevalence and sociodemographic correlates of food insecurity among post-secondary students and non-students of similar age in Canada
Source: BMC Public Health. 2023 May 25;23:954. doi: 10.1186/s12889-023-15756-y (PMC10209945; doi:10.1186/s12889-023-15756-y)
Supplement: Supplementary file 1 — Supplementary Material 1 [file 12889_2023_15756_MOESM1_ESM.docx]

**Additional file 1: Cost of Learning Index and the grouping of provinces**

|  | **Cost of Learning Index (descending)^1^** | | | **Expected tuition and compulsory fees ($) 2016/17^1^** | **Actual tuition and compulsory fees ($) 2017/18^2^** | **Annual increase rate (%) of tuition and compulsory fees from 2012-2018** | **Grouping according to 2016-17e Cost of Learning Index** | **Grouping according to actual tuition and compulsory fees for 2017/18** |
| --- | --- | --- | --- | --- | --- | --- | --- | --- |
|  | **2012–2013** | **2013–2014e^3^** | **2016–2017e** |  |  |  |  |  |
| Newfoundland and Labrador | 126 | 125 | 118 | 2,886 | 3,659 | 5.57 | High affordability | High affordability |
| Quebec | 127 | 129 | 132 | 4,086 | 3,744 | 2.64 | High affordability | High affordability |
| Manitoba | 183 | 184 | 182 | 4,441 | 5,024 | 3.89 | High affordability | High affordability |
| Prince Edward Island | 208 | 218 | 246 | 7,293 | 7,132 | 3.63 | Medium affordability | Medium affordability |
| British Columbia | 246 | 248 | 251 | 6,129 | 6,400 | 3.22 | Medium affordability | Medium affordability |
| Nova Scotia | 265 | 272 | 285 | 7,574 | 8,546 | 5.81 | Medium affordability | Low affordability* |
| New Brunswick | 280 | 285 | 291 | 7,094 | 7,476 | 3.46 | Medium affordability | Medium affordability |
| Ontario | 280 | 290 | 311 | 9,517 | 9,450 | 4.05 | Low affordability | Low affordability |
| Saskatchewan | 285 | 295 | 325 | 7,912 | 7,669 | 3.32 | Low affordability | Low affordability |
| Alberta | 298 | 298 | 331 | 8,077 | 6,822 | 0.71 | Low affordability | Medium affordability* |

^1^From MacDonald et al, 2013.

^2^From Statistics Canada, 2016.

^3^”e” indicates estimated values (MacDonald et al, 2013).

**Changes in the classification of two provinces for purposes of this study:**

Nova Scotia was moved to the low affordability group and Alberta was moved to the medium affordability group for the following reasons:

1. Although the Cost of Learning index was calculated based on multiple factors, the amount of tuition and compulsory fees seems to be one of the most important indicators. The use of tuition and compulsory fees predicts the same results as the Cost of Learning Index when the provinces are grouped into low, medium and high affordability groups.
2. Alberta had a three-year tuition freeze from 2015-2018, which could make it more affordable than expected (Expected fees of 2016/17 from CCPA: $8077, 2^nd^ highest within Canada; actual fees from Stats CA in 2017/18: $6822, 6^th^ highest within Canada). Alberta also has the lowest annual increase rate of 0.71%.
3. Nova Scotia has the highest annual tuition (and compulsory fees) increase rate of 5.81%, and by 2018, it had the 2^nd^ highest tuition among all provinces, as compared to the 4^th^ highest predicted by CCPA (Expected: $7574; Actual: $8127).

**References:**

Macdonald D, Shaker E, Wodrich N: Degrees of Uncertainty. Navigating the Changing Terrain of University Finance. In.: Canadian Centre for Policy Alternatives; 2013.

Statistics Canada. Canadian students, tuition and additional compulsory fees, by level of study [Internet]. 2016 [cited 2022 Apr 22]. Available from: https://www150.statcan.gc.ca/t1/tbl1/en/tv.action?pid=3710012101
