# Supplementary material for: Prevalence and sociodemographic correlates of food insecurity among post-secondary students and non-students of similar age in Canada
Source: BMC Public Health. 2023 May 25;23:954. doi: 10.1186/s12889-023-15756-y (PMC10209945; doi:10.1186/s12889-023-15756-y)
Supplement: Supplementary file 2 — Supplementary Material 2 [file 12889_2023_15756_MOESM2_ESM.docx]

**Additional File 2: Socio-demographic characteristics of the sample, stratified by student status and food insecurity status** **(n = 11,679).**

|  | **Full-time post-secondary Students** | | **Part-time post-secondary students** | | **Non-students** | |
| --- | --- | --- | --- | --- | --- | --- |
|  | **Food secure** | **Food insecure** | **Food secure** | **Food insecure** | **Food secure** | **Food insecure** |
| **Overall %** | 85.0% | 15.0% | 83.8% | 16.2% | 80.9% | 19.1% |
| **Provincial ranking of affordability of post-secondary schooling, %** |  |  |  |  |  |  |
| High affordability (Newfoundland and Labrador, Quebec, Manitoba) | 83.6% | 16.4% | 87.0% | 13.0% | 82.7% | 17.3% |
| Medium affordability (Prince Edward Island, New Brunswick, Alberta, British Columbia) | 86.5% | 13.5% | 76.4% | 23.6% | 78.2% | 21.8% |
| Low affordability (Nova Scotia, Ontario, Saskatchewan) | 84.9% | 15.1% | 86.0% | 14.0% | 81.5% | 18.5% |
| **Age groups, %** |  |  |  |  |  |  |
| 19-24 | 85.5% | 14.5% | 84.2% | 15.8% | 75.2% | 24.8% |
| 25-30 | 83.1% | 16.9% | 83.5% | 16.5% | 83.3% | 16.7% |
| **Sex** |  |  |  |  |  |  |
| Male | 85.5% | 14.5% | 88.3% | 11.7% | 81.4% | 18.6% |
| Female | 84.5% | 15.5% | 80.5% | 19.5% | 80.3% | 19.7% |
| **Immigration status, %** |  |  |  |  |  |  |
| Canadian born | 85.7% | 14.3% | 84.9% | 15.1% | 80.1% | 19.9% |
| Immigrant ≤ 5 years | 76.3% | 23.7% | 66.6% | 33.4% | 82.9% | 17.1% |
| Immigrant > 5 years | 83.8% | 16.2% | 86.2% | 13.8% | 84.9% | 15.1% |
| **Aboriginal Status, %** |  |  |  |  |  |  |
| Non-aboriginal | 85.2% | 14.8% | 84.3% | 15.7% | 81.6% | 18.4% |
| Aboriginal | 74.6% | 25.4% | 68.6% | 31.4% | 63.5% | 36.5% |
| **Highest level of education, %** |  |  |  |  |  |  |
| High school or less | 84.1% | 15.9% | 76.7% | 23.3% | 72.2% | 27.8% |
| Some post-secondary education (no certificate) | 81.8% | 18.2% | 78.0% | 22.0% | 72.7% | 27.3% |
| Post-secondary certificate below Bachelor's | 85.7% | 14.3% | 86.6% | 13.4% | 81.9% | 18.1% |
| Bachelor's degree or above | 89.6% | 10.4% | 88.6% | 11.4% | 91.1% | 8.9% |
| **Size of area of residence, %** |  |  |  |  |  |  |
| Rural area/ population < 100,000 | 87.0% | 13.0% | 85.1% | 14.9% | 78.5% | 21.5% |
| Population 100,000- 499,999 | 83.0% | 17.0% | 78.0% | 22.0% | 80.7% | 19.3% |
| Population ≥ 500,000 | 84.7% | 15.3% | 84.6% | 15.4% | 82.4% | 17.6% |
| **Living arrangements, %** |  |  |  |  |  |  |
| Living alone with/without roommates | 80.2% | 19.8% | 78.5% | 21.5% | 79.1% | 20.9% |
| Living with families (parents or relatives) | 87.1% | 12.9% | 89.2% | 10.8% | 80.9% | 19.1% |
| Living with spouse, no children | 83.9% | 16.1% | 81.6% | 18.4% | 87.5% | 12.5% |
| Living with children | 65.6% | 34.4% | 54.7% | 45.3% | 74.0% | 26.0% |
| **After-tax income, adjusted for household size (Mean ± SE)** | $54152 ± 1075.3 | $31307 ± 1253.6 | $59255 ± 1864.8 | $39290 ± 2491.9 | $57552 ± 588.9 | $38945 ± 852.6 |
| **Major source of economic family income, %** |  |  |  |  |  |  |
| Wages, salaries, or self-employment | 86.8% | 13.2% | 85.5% | 14.5% | 82.7% | 17.3% |
| Social assistance | 32.4% | 67.6% | * | * | 24.4% | 75.6% |
| Other income sources | 79.5% | 20.5% | 71.2% | 28.8% | 74.6% | 25.4% |
| Ownership of dwelling, % |  |  |  |  |  |  |
| Owned by a member of the household | 90.4% | 9.6% | 87.5% | 12.5% | 85.5% | 14.5% |
| Not owned by a member of the household | 75.4% | 24.6% | 77.4% | 22.6% | 74.6% | 25.4% |
| **Received scholarship, %** |  |  |  |  |  |  |
| Yes | 83.5% | 16.5% | 68.7% | 31.3% | NA | NA |
| No | 85.4% | 14.6% | 85.1% | 14.9% | NA | NA |
| **Employment, %** |  |  |  |  |  |  |
| Full-year full-time worker | 81.9% | 18.1% | 90.5% | 9.5% | NA | NA |
| Part-time worker | 86.2% | 13.8% | 79.8% | 20.2% | NA | NA |
| Did not work during the reference year | 81.6% | 18.4% | 70.8% | 29.2% | NA | NA |

*Cell sizes were not releasable as per RDC guidelines.

^a^Include government transfers except social assistance (child benefits, Canada Pension Plan (CPP)/Quebec Pension Plan (QPP) benefits, Old Age Security (OAS) and Guaranteed Income Supplement (GIS)/Spouse’s Allowance, employment insurance benefits, workers’ compensation benefits, GST/HST credit, provincial tax credits, and other government transfers), investment income, retirement pensions, other income, or no income during the reference year.

^b^NA: not applicable.
